# A comparison of synthetic oligodeoxynucleotides, DNA fragments and AAV-1 for targeted episomal and chromosomal gene repair

**DOI:** 10.1186/1472-6750-9-35

**Published:** 2009-04-20

**Authors:** Xavier Leclerc, Olivier Danos, Daniel Scherman, Antoine Kichler

**Affiliations:** 1 Genethon-FRE3087 CNRS-Université d'Evry Val d'Essonne, 1 rue de l'Internationale, Département de Recherche Exploratoire, BP 60, 91002 – Evry Cedex, France; 2 Inserm U781 and Université Paris Descartes, Hôpital Necker Enfants Malades, 149 rue de Sèvres, 75015 – Paris, France

## Abstract

**Background:**

Current strategies for gene therapy of inherited diseases consist in adding functional copies of the gene that is defective. An attractive alternative to these approaches would be to correct the endogenous mutated gene in the affected individual. This study presents a quantitative comparison of the repair efficiency using different forms of donor nucleic acids, including synthetic DNA oligonucleotides, double stranded DNA fragments with sizes ranging from 200 to 2200 bp and sequences carried by a recombinant adeno-associated virus (rAAV-1). Evaluation of each gene repair strategy was carried out using two different reporter systems, a mutated eGFP gene or a dual construct with a functional eGFP and an inactive luciferase gene, in several different cell systems. Gene targeting events were scored either following transient co-transfection of reporter plasmids and donor DNAs, or in a system where a reporter construct was stably integrated into the chromosome.

**Results:**

In both episomal and chromosomal assays, DNA fragments were more efficient at gene repair than oligonucleotides or rAAV-1. Furthermore, the gene targeting frequency could be significantly increased by using DNA repair stimulating drugs such as doxorubicin and phleomycin.

**Conclusion:**

Our results show that it is possible to obtain repair frequencies of 1% of the transfected cell population under optimized transfection protocols when cells were pretreated with phleomycin using rAAV-1 and dsDNA fragments.

## Background

The conventional approach for treatment of genetic disorders by gene therapy is gene addition that consists of supplying a functional cDNA copy of the defective gene. This can be achieved by delivering the desired DNA sequences to the target cells using either viral or non viral methods. An alternative to this strategy is to correct the endogenous mutated gene in the affected individual through gene repair. In principal, genetic repair strategies have significant therapeutic and safety advantages over the traditional cDNA gene therapy approaches when treating inherited diseases [1]. A targeting approach would extend the possibility of therapeutic correction to both recessive and dominant diseases [2]. The corrected gene would be under the control of its cognate control sequences, ensuring cell-specific and appropriate level and duration of expression [3]. Gene targeting is expected to be stable and would minimize limitations due to the size of the gene to be corrected as well as the risk linked to insertional mutagenesis. Last, short DNA fragments or synthetic oligonucleotides are often sufficient as donor sequences and are likely to be more efficiently routed into the nucleus than large plasmid DNA constructs used in gene replacement approaches [4].

Several gene repair strategies were developed over the last two decades, including the use of single- [5,6] and double-stranded DNA fragments [7-11], small single-stranded oligonucleotides [12-14], RNA-DNA chimeras [15-17] and triple-helix forming oligonucleotides [18,19]. As it is the case for plasmid DNA, these nucleic acid-based repair elements can be introduced by non viral delivery methods. Another repair approach that has been described consists of using recombinant adeno-associated virus (rAAV) [20,21] or lentiviral vectors [22]. Most of these strategies displayed low but detectable activity in cell lines. In other studies, gene modification was reported in primary and transformed cells [9,23], in hematopoietic progenitor cells [24,25] or in animal models such as the CFTR mouse and dystrophic mice and dogs [16,26-28]. Although high correction frequencies have been reported, in particular by using RNA/DNA chimeraplasts [29,30], they remain the subject of controversy [31-34]. Most often, targeted alteration of genomic DNA in mammalian cells occurs at frequencies that are only detectable by highly sensitive assays.

A current problem has been a difficulty in comparing the repair frequencies obtained by different methods, because the experimental conditions are rarely identical (e.g., differences in the target gene, the cell line, the transfection method). By taking this into consideration, the studies presented here compare gene correction efficiency of modified and non-modified single-stranded oligodeoxynucleotides (ssODN) to linear double-stranded DNA (dsDNA) fragments and a recombinant AAV-1 vector using both episomal and chromosomal targets. Further, we have assessed whether pre-treatment of target cells with drugs that stimulate DNA repair, such as doxorubicin and phleomycin, can increase gene correction. The data indicate that dsDNA fragments or rAAV-1 result in chromosomal repair frequencies of ~1% when the cells are pretreated with phleomycin.

## Results

### Generation and characterization of the mutated reporter constructs

**Figure 1:**
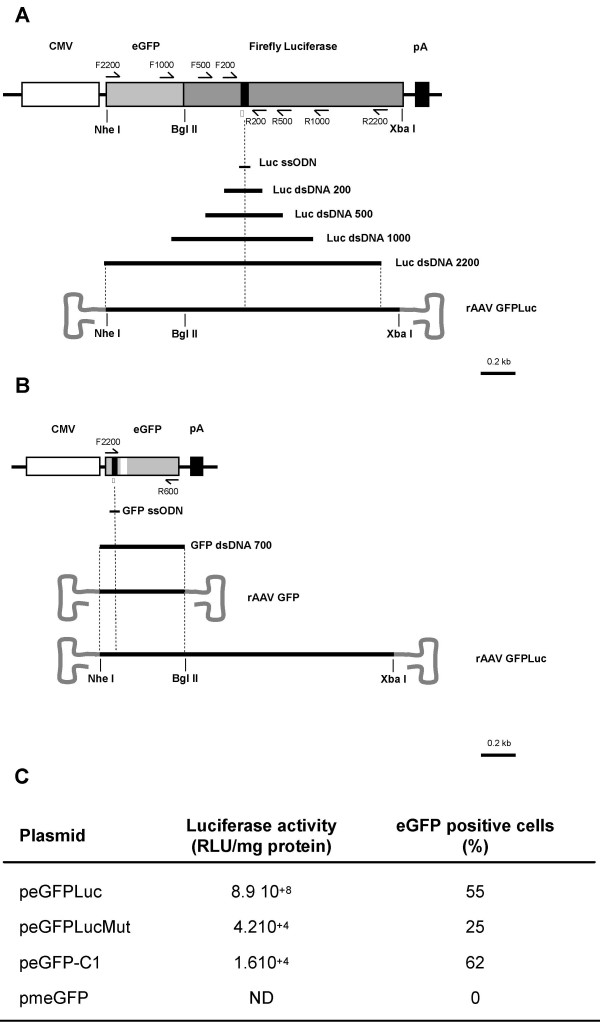
**Reporter constructs and correction agents**. (A) Representation of the peGFPLucMut plasmid. A premature stop codon (black plain bar) was introduced into the luciferase portion of the eGFP-Luciferase fusion gene driven by a CMV promoter. (B) In the pmeGFP plasmid, the eGFP gene harbors a premature stop codon (black plain bar) and a silent mutation (white bar) used as tag sequence. The following information are given for the repair target vectors: the asterisk indicates the position of the mutation to correct and the half-arrows represent the location of the primers used to produce the dsDNA fragments. The length of homology between the gene repair agents and the targeted sequences are represented below each vector. Sequence of the primers used to generate the dsDNA fragments were as follows: F2200, 5'-AAATGTCGTAACAACTCCGCC-3'; F1000, 5'-TACAACTACAACAGCCACAAC-3'; F500, 5'-CGCCAAAAACATAAAGAAAGG-3'; F200, 5'-GCTATGAAGAGATACGCCCT-3'; R2200, 5'-AATGTAGCCATCCATCCTTGTC-3'; R1000, 5'-AATCTCACGCAGGCAGTTC-3'; R500, 5'-CGAACGTGTACATCGACTG-3'; R200, 5'-CAACACCGGCATAAAGAATTG-3' and R700, 5'-TGCTCAGGTAGTGGTTGTCG-3. CMV, immediate-early cytomegalovirus promoter (white box); eGFP gene (light grey box); Luciferase gene (dark grey box); pA, SV40 early mRNA polyadenylation box. BglII and XbaI, location of the BglII and XbaI restriction sites. (C) Luciferase activity and transfection efficiency expressed as a percentage obtained with HEK293T cells using different plasmids.

Since gene repair frequencies are often difficult to assess either because of their frequency or because there is not a selectable assay system, highly sensitive assays are a major requirement. Although sensitive, PCR-based assays can lead to artefacts if the experiments are incorrectly performed [35-37]. In contrast, reporter genes provide a convenient means to evaluate gene targeting efficacy. The *luciferase *gene encodes a protein that produces light through an enzymatic reaction and is particularly attractive because it is sensitive and can be easily quantified. Repairing a mutated gene instead of inducing a mutation is preferable because it is easier to measure an increase in function above background than to detect a small decrease in a high level of activity [38]. The peGFPLucMut plasmid (Figure 1A) has a premature stop codon generated by a single nucleotide change in the open reading frame, upstream of the luciferase enzyme catalytic site. To confirm that the mutation introduced inactivates luciferase activity and does not affect the fluorescence properties of eGFP, the peGFPLucMut plasmid was compared to the wild-type expression cassette peGFPLuc (Figure 1C). Therefore, HEK293T cells were transfected with both plasmids and were analysed 40 h post-transfection for luciferase and GFP activity. A > 2 × 10^4^-fold reduction in luciferase activity was observed with the mutant construct as compared to the wild-type plasmid (Figure 1C). Flow cytometric analysis showed that inactivation of the luciferase gene only resulted in a 2- to 3-fold decrease of both the number of eGFP positive cells (Figure 1C) and in the mean fluorescence (not shown). This minor effect of the luciferase mutation on the activity of the upstream eGFP coding sequences could be due to mRNA destabilization caused by the premature stop codon, through the Nonsense-Mediated Decay pathway [39].

A second target vector was developed to estimate the percentage of cells in which repair occurred. To this end, a point mutation that causes a premature stop codon was introduced into the eGFP open reading frame of the peGFP-C1 plasmid, creating the pmeGFP plasmid (Figure 1B) and abolishing GFP fluorescence [40] (Figure 1C).

### Episomal gene correction with single-stranded oligodeoxynucleotides

**Figure 2:**
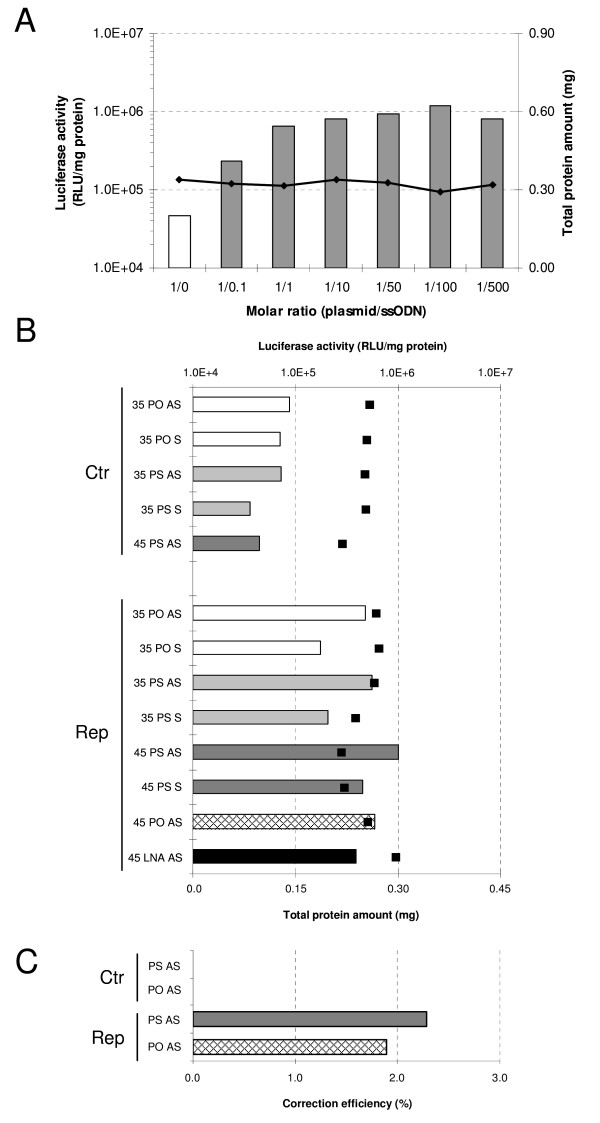
**Episomal ssODN-mediated gene repair assay**. (A) Dose-curve for molar ratios of plasmid/Luc PO AS 45-mer. Total protein content (black diamonds) is shown on the right y-axis. (B) HEK293T cells were transfected with both, Luc targeting oligonucleotides and the repair target vector peGFPLucMut using the molar ratio plasmid/gene repair agent of 1/100. Two days after, cell samples were submitted to luciferase activity measurement to quantify the gene repair efficiency (left y-axis). Total protein content is indicated with the black squares. (C) Similar experiments as in (B) were conducted with the eGFP targeted oligonucleotides and the mutated pmeGFP using the molar ratio plasmid/gene repair agent of 1/100. The repair was followed by eGFP positive cells counting using flow cytometry. Luc, luciferase; GFP, green fluorescent protein; AS, antisense; S, sense; PO, phosphodiester; PS, phosphorothioate; LNA, locked nucleic acid; 35, 35mer; 45, 45mer; Ctr, non-correcting control oligonucleotide; Rep, repair oligonucleotide.

Episomal gene repair assays were performed with ssODNs designed to target the point mutations introduced into the two reporter genes. Preliminary studies were conducted to determine the optimal transfection conditions and target/ssODN ratio to achieve efficient gene repair. In HEK293T cells, Lipofectamine mixed with 1.75 μg/well of target peGFPLucMut plasmid and amounts of ssODN corresponding to a molar ratio (target plasmid/ssODN) of 1/100 gave the highest gene correction efficiency (Figure 2A). Using these conditions, the transfection efficiency measured with the peGFPLucMut construct reproducibly ranged between 18 and 25%.

We then compared the ability of modified and non-modified ssODNs to repair the point mutation carried by the luciferase gene of the peGFPLucMut plasmid. Notably, all antisense repair Luc ssODNs harbor the same nucleotidic sequence and were designed to hybridize the mutated eGFPLuc in such a way that the correcting base is positioned in the center of the nucleotide chain of 35 and 45 residues (Table 1). When transfected, the 35-mer PO-AS control oligonucleotide (Luc PO-AS Ctr) generated luciferase activity levels that were similar to those obtained with the peGFPLucMut plasmid alone. This indicates that the control ODN does not induce repair of the point mutation, as expected. By contrast, co-transfection of the mutated plasmid with the repair 35-mer PO-AS oligonucleotide (Luc PO-AS Rep) resulted in reporter gene activity that were 5-times higher than control levels (Figure 2B). Despite the advantageous sensitivity of the luciferase system, it is still difficult to determine the percentage of cells which have undergone gene correction. To address this question, we used the pmeGFP plasmid as target for the episomal gene correction assays employing oligonucleotides (Table 1) designed to target the premature stop codon. By using the same experimental conditions as with the peGFPLucMut targeting, control and antisense repair oligonucleotides (35 nucleotides) were compared. Notably, the ODN sequence used was previously found to be the most efficient for repair of pmeGFP [40]. As shown in Figure 2C, the correcting ODNs generated ~2% GFP positive cells while the control ODNs did not lead to detectable levels of GFP expression. These results clearly indicate that the tools and experimental conditions we developed allowed us to detect DNA repair events.

**Table 1:** List of the single-stranded oligodeoxynucleotides used to target the mutated genes

**Name**	**Size**	**Sequence**	**Characteristics**
Luc PO AS Ctr	35 b	5'-TGATTTGTATTCAGCCC**C**TATCGTTTCATAGCTTC-3'	Antisense Control phosphodiester ssODN

Luc PO S Ctr	35 b	5'-GAAGCTATGAAACGATA**G**GGGCTGAATACAAATCA-3'	Sense Control phosphodiester ssODN

Luc PS AS Ctr	35 b	5'-TsGsAsTTTGTATTCAGCCC**C**TATCGTTTCATAGCsTsTsC-3'	Antisense Control ssODN with 3 PS linkages at each end

Luc PS S Ctr	35 b	5'-GsAsAsGCTATGAAACGATA**G**GGGCTGAATACAAAsTsCsA-3'	Sense Control ssODN with 3 PS linkages at each end

Luc PS AS Ctr	45 b	5'-TsTsCsTGTGATTTGTATTCAGCCC**C**TATCGTTTCATAGCTTCTGsCsCsA-3'	Antisense Control ssODN with 3 PS linkages at each end

Luc PO AS Rep	35 b	5'-TGATTTGTATTCAGCCC**A**TATCGTTTCATAGCTTC-3'	Antisense phosphodiester ssODN

Luc PO S Rep	35 b	5'-GAAGCTATGAAACGATA**T**GGGCTGAATACAAATCA-3'	Sense phosphodiester ssODN

Luc PS AS Rep	35 b	5'-TsGsAsTTTGTATTCAGCCC**A**TATCGTTTCATAGCsTsTsC-3'	Antisense ssODN with 3 PS linkages at each end

Luc PS S Rep	35 b	5'-GsAsAsGCTATGAAACGATA**T**GGGCTGAATACAAAsTsCsA-3'	Sense ssODN with 3 PS linkages at each end

Luc PO AS Rep	45 b	5'-TTCTGTGATTTGTATTCAGCCC**A**TATCGTTTCATAGCTTCTGCCA-3'	Antisense phosphodiester ssODN

Luc PS AS Rep	45 b	5'-TsTsCsTGTGATTTGTATTCAGCCC**A**TATCGTTTCATAGCTTCTGsCsCsA-3'	Antisense ssODN with 3 PS linkages at each end

Luc PO S Rep	45 b	5'-TsGsGsCAGAAGCTATGAAACGATA**T**GGGCTGAATACAAATCACAsGsAsA-3'	Sense ssODN with 3 PS linkages at each end

Luc LNA 1.1 AS Rep	45 b	5'-TTCTGTGATTTGTATTCAGCCC**A**TATCGTTTCATAGCTTCTGCCA-3'	Antisense ssODN with 1 LNA residue at each end

Luc LNA 4.4 AS Rep	45 b	5'-TTCTGTGATTTGTATTCAGCCC**A**TATCGTTTCATAGCTTCTGCCA-3'	Antisense ssODN with 4 LNA residues at each end

Luc LNA 4r AS Rep	45 b	5'-TTCTGTGATTTGTATTCAGCCC**A**TATCGTTTCATAGCTTCTGCCA-3'	Antisense ssODN with 4 LNA residues distributed over the sequence

GFP PO AS Ctr	35 b	5'-TGGTCACGAGGGTTGGC**T**AGGGCACGGGCAGCTTG-3'	Antisense Control phosphodiester ssODN

GFP PS AS Ctr	35 b	5'-TsGsGsTCACGAGGGTTGGC**T**AGGGCACGGGCAGCsTsTsG-3'	Antisense Control ssODN with 3 PS linkages at each end

GFP PO AS Rep	35 b	5'-TGGTCACGAGGGTTGGC**C**AGGGCACGGGCAGCTTG-3'	Antisense phosphodiester ssODN

GFP PS AS Rep	35 b	5'-TsGsGsTCACGAGGGTTGGC**C**AGGGCACGGGCAGCsTsTsG-3'	Antisense ssODN with 3 PS linkages at each end

Luc, luciferase; GFP, green fluorescent protein; AS, antisense; S, sense; Ctr, non-correcting control oligonucleotide; Rep, repair oligonucleotide; PO, phosphodiester; PS, phosphorothioate; LNA, locked nucleic acid (underlined); s, phosphorothioate linkage; in bold, position of the base to correct.

Furthermore, oligonucleotides containing different modifications were designed and evaluated (Table 1). The study compared the gene correction potential of antisense 35- and 45-mer ssODNs harboring (Luc PS-AS Rep) or not (Luc PO-AS Rep) three phosphorothioate linkages at each end. These modifications were included to protect the oligonucleotides from exonuclease degradation. The results show that the repair efficiency is not significantly higher with the PS-modified ODNs (Figure 2B). This was also the case when comparing the PS ODN designed to repair the pmGFP to the PO ODN (Figure 2C). Transfection of the 35- and 45-mer antisense ODNs generated higher luciferase activities than their sense counterparts, indicating a strand bias in repair of episomal gene targets [14]. It is also interesting to note that the addition of 10 nucleotides to the ODNs results in an insignificant modification of the repair capacity (comparison between 35- and 45-mer ODNs).

Locked nucleic acids (LNAs) residues within the repair fragment were also examined in terms of their ability to improve the efficiency of repair. These residues have a methylene bridge connecting the 2'-oxygen with the 4'-carbon of ribose. The rationale for including such residues lies in the fact that LNAs were shown to display a higher affinity to DNA residues than DNA itself, and therefore may stabilize the ODN/DNA interactions and result in a higher frequency of repair [41,42]. The effect of including LNA residues was studied with 3 ODNs: two had the LNA residues at each end (4 and 1 residues at each end for LNA 4.4 and LNA 1.1, respectively). The third contains four LNAs residues distributed over the whole sequence (Table 1). Notably, the Tm measurements showed that LNA 4.4 and LNA 4r, increased the Tm by 3–4°C; whereas the Tm remained unchanged as compared to the PO oligonucleotides when there was 1 LNA residue introduced at each end of the ODN. *In vitro *results show that, although the 3 antisense oligonucleotides mediate gene repair, none was more efficient than the PS or PO ODNs (Figure 2C and data not shown). It is also worth noting that there is no difference between oligonucleotides harboring one or four LNA residues at the extremities. In addition, the presence of an LNA residue near the target base unfavorably affects the correction efficiency as was indicated with the Luc LNA 4r AS ODN (data not shown).

### Episomal gene correction with double-stranded DNA fragments

**Figure 3:**
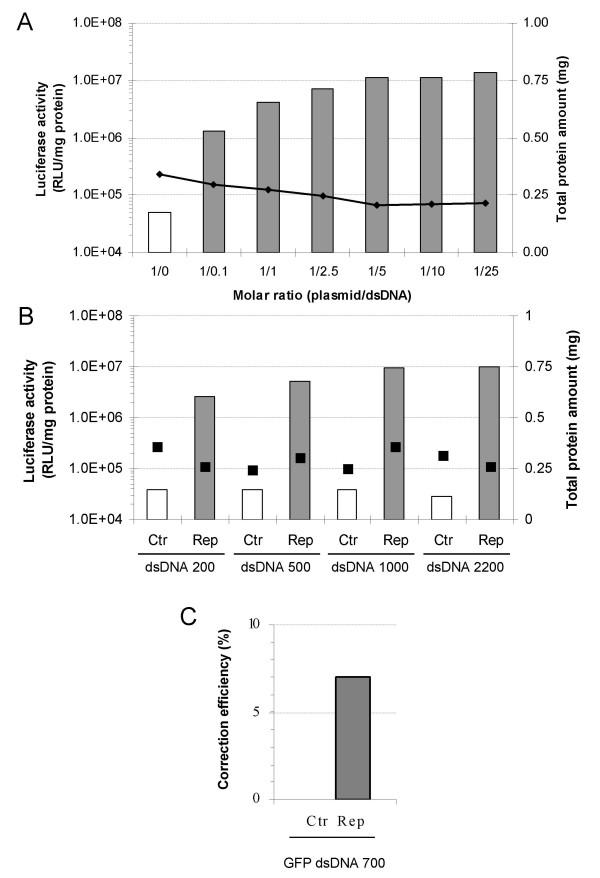
**Episomal dsDNA-mediated gene repair**. (A) Dose-curve for molar ratios of plasmid/1000 ds DNA Rep. Total protein content (black diamonds) is shown on the right y-axis (B) HEK293T cells were transfected with Luc dsDNA fragments and the target vector peGFPLucMut using the molar ratio plasmid/gene repair agent of 1/10 (plain bars). Two days after, cell samples were submitted to luciferase activity measurement to quantify the gene repair efficiency (left y-axis). Total protein content (black squares) is shown on the right y-axis. (C) Similar experiments than in (B) were conducted with the GFP targeted dsDNA fragment and the pmeGFP vector using the molar ratio plasmid/gene repair agent of 1/10 (plain bars). The repair efficiency was measured by eGFP positive cells counting using flow cytometry. For each dsDNA fragments: Ctr, non-correcting control fragment; Rep, repair dsDNA fragment.

To evaluate the impact of DNA fragment size on the repair efficiency, dsDNA fragments with sizes ranging from 200 to 2200 bp were generated. As for the ssODNs, the fragments were generated such that they were able to anneal with the eGFPLuc ORF (Table 2) and that the target base was placed in the center of the fragment. This latter point is important since previous studies have indicated that the frequency of homologous recombination is dependent on the length of homologous sequence flanking each side of the mutated site [43]. Care was also taken to ensure that the DNA fragments did not overlap flanking regulatory sequences such as the promoter region (Figure 1A). This is necessary to eliminate the possibility that the resulting luciferase expression was not due to spurious activation of the gene. For each size, the repair (Rep) and control (Ctr) fragments were compared for their repair efficiency. Using the same transfection conditions as with ssODNs, the target/dsDNA fragment ratio resulting in the highest gene correction efficiency was 1/10 (Figure 3A). As with the ssODNs, the plasmid transfection efficiency ranged between 18 and 25%. The episomal repair assays with the different DNA fragments gave the following results: 1) transfection using the repair DNA fragments resulted in luciferase expression levels that were at least two orders of magnitude higher than those obtained with the control fragments (Figure 3B), 2) the frequency of repair increases slightly with the size of the repairing DNA fragment (Rep), with a plateau size of 500 bp, and 3) the data shows that dsDNA-mediated repair is about ten-fold more efficient than ssODNs (comparing the results of Figure 2A and 3A, generated in the same experiment).

**Table 2:** List of the double-stranded DNA fragments used to target the mutated genes

**Name**	**Size**	**Sequence**
200 dsDNA Ctr	195 bp	5'-CGGCA..//..TGGCAGAAGCTATGAAACGATA**G**GGGCTGAATACAAATCACAGAA..//..TGTTG-3' 3'-GCCGT..//..ACCGTCTTCGATACTTTGCTAT**C**CCCGACTTATGTTTAGTGTCTT..//..CAAGC-5'

500 dsDNA Ctr	494 bp	5'-CACGT..//..TGGCAGAAGCTATGAAACGATA**G**GGGCTGAATACAAATCACAGAA..//..GTTCG-3' 3'-GTGCA..//..ACCGTCTTCGATACTTTGCTAT**C**CCCGACTTATGTTTAGTGTCTT..//..CAAGC-5'

1000 dsDNA Ctr	980 bp	5'-TACAA..//..TGGCAGAAGCTATGAAACGATA**G**GGGCTGAATACAAATCACAGAA..//..AGATT-3' 3'-ATGTT..//..ACCGTCTTCGATACTTTGCTAT**C**CCCGACTTATGTTTAGTGTCTT..//..TCTAA-5'

2200 dsDNA Ctr	2133 bp	5'-AAATG..//..TGGCAGAAGCTATGAAACGATA**G**GGGCTGAATACAAATCACAGAA..//..ACATT-3' 3'-TTTAC..//..ACCGTCTTCGATACTTTGCTAT**C**CCCGACTTATGTTTAGTGTCTT..//..TGTAA-5'

200 dsDNA Rep	195 bp	5'-CGGCA..//..TGGCAGAAGCTATGAAACGATA**T**GGGCTGAATACAAATCACAGAA..//..TGTTG-3' 3'-GCCGT..//..ACCGTCTTCGATACTTTGCTAT**A**CCCGACTTATGTTTAGTGTCTT..//..CAAGC-5'

500 dsDNA Rep	494 bp	5'-CACGT..//..TGGCAGAAGCTATGAAACGATA**T**GGGCTGAATACAAATCACAGAA..//..GTTCG-3' 3'-GTGCA..//..ACCGTCTTCGATACTTTGCTAT**A**CCCGACTTATGTTTAGTGTCTT..//..CAAGC-5'

1000 dsDNA Rep	980 bp	5'-TACAA..//..TGGCAGAAGCTATGAAACGATA**T**GGGCTGAATACAAATCACAGAA..//..AGATT-3' 3'-ATGTT..//..ACCGTCTTCGATACTTTGCTAT**A**CCCGACTTATGTTTAGTGTCTT..//..TCTAA-5'

2200 dsDNA Rep	2133 bp	5'-AAATG..//..TGGCAGAAGCTATGAAACGATA**T**GGGCTGAATACAAATCACAGAA..//..ACATT-3' 3'-TTTAC..//..ACCGTCTTCGATACTTTGCTAT**A**CCCGACTTATGTTTAGTGTCTT..//..TGTAA-5'

GFP 700 dsDNA Ctr	732 bp	5'-AAATG..//..CAAGCTGCCCGTGCCCT**A**GCCAACCCTCGTGACCA..//..GAGCA-3' 3'-TTTAC..//..GTTCGACGGGCACGGGA**T**CGGTTGGGAGCACTGGT..//..CTCGT-5'

GFP 700 dsDNA Rep	732 bp	5'-AAATG..//..CAAGCTGCCCGTGCCCT**G**GCCAACCCTCGTGACCA..//..GAGCA-3' 3'-TTTAC..//..GTTCGACGGGCACGGGA**C**CGGTTGGGAGCACTGGT..//..CTCGT-5'

Ctr, non-correcting control fragment; Rep, repair fragment; GFP, green fluorescent protein; in bold, position of the base to correct.

To quantify the percentage of cells repaired, a 700 bp dsDNA fragment was generated using the wild-type and the mutant eGFP genes as templates, respectively (Table 2). Figure 3C shows that about 7% of the entire cell population was eGFP positive after repair. When compared to the ssODN PO-AS, the results indicated that the dsDNA fragment was significantly more efficient in repairing the point mutation than the ssODN (Figure 2C and 3C).

### Episomal gene correction with rAAV-1

**Figure 4:**
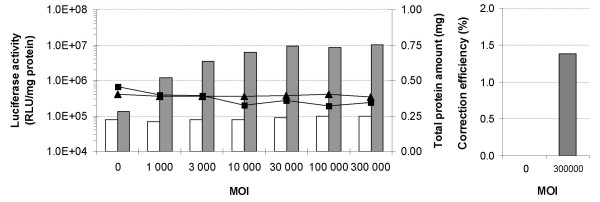
**Episomal rAAV-mediated gene repair**. (A) HEK293T cells were infected with an increasing multiplicity of infection (MOI) of the rAAV2/1-GFPLuc vector following (dark grey bars) or not (white bars) a transfection step with the target vector peGFPLucMut. Two days after, cell samples were submitted to luciferase activity measurement to quantify the gene repair efficiency (left y-axis). Total protein content is shown on the right y-axis (squares for repair conditions and triangles for the control experiments). (B) Similar experiment as in (A) conducted with the target vector pmeGFP followed by eGFP positive cells counting to determine the repair frequency.

Adeno-associated virus (AAV) is a single-stranded, linear DNA virus with a 4.7 kb genome consisting of the viral *rep *and *cap *genes flanked by inverted terminal repeat (ITR) sequences. AAV vectors containing foreign DNA between the ITRs (Figure 1) can be packaged by *rep *and *cap *gene products supplied in *trans*. By using this methodology, a rAAV-GFPLuc vector pseudotyped with serotype 1 capsid proteins (rAAV1) was generated and then used in the episomal gene correction assays. It contains a 2.4 kb eGFPLuc fragment that is only a little larger than the 2.2 kb fragment used in the dsDNA-mediated repair assays. The fragment is flanked by the two inverted terminal repeats of AAV serotype 2 (Figure 1). Use of this recombinant AAV vector allows direct comparison of the repair efficiency between transfected dsDNA and the rAAV-1 vector. Preliminary studies were conducted to determine the optimal transfection and infection conditions for efficient gene repair. In HEK293T cells, transfection of 1.75 μg of peGFPLucMut followed by rAAV infection gave the highest efficiency (data not shown). By using these optimized conditions and multiplicities of infection (MOI) from 1 000 to 300 000, the ability of rAAV-GFPLuc vectors to restore luciferase activity to the mutant reporter construct was evaluated. Infection of non-transfected cells with the rAAV-GFPLuc vectors resulted in luciferase levels similar to those obtained with cells transfected with the target plasmids alone. However, addition of rAAV-GFPLuc to transfected cells showed a significant increase of the luciferase activity (Figure 4A). At MOIs = of 3000, the luciferase levels were comparable to those observed with dsDNA fragments (Figure 3B and 4). Although not fully homologous to the gene to repair due to the presence of about 1650 nt of the luciferase gene, correction of pmeGFP transfected HEK293T cells by rAAV GFPLuc resulted in 1.38% GFP positive cells (Figure 4B). Notably, a similar efficiency was obtained with the rAAV-GFP vector (Figure 1B), indicating that the luciferase segment of the rAAV-GFPLuc did not negatively interfere with the repair process (data not shown).

### Drug-enhanced DNA repair

**Figure 5:**
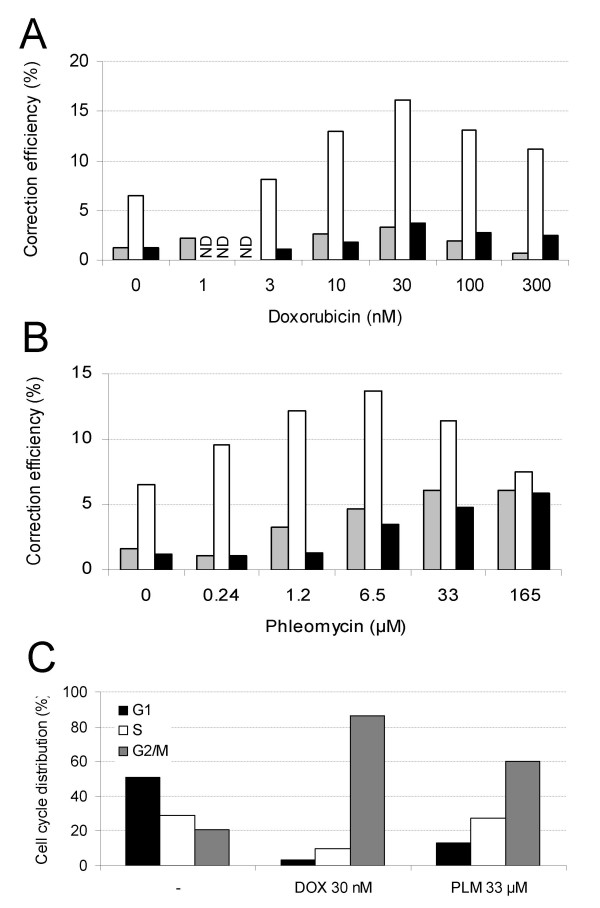
**Doxorubicin and phleomycin stimulation of ssODN-, dsDNA-, and rAAV-mediated episomal gene repair**. (A) HEK293T cells were treated with increasing doses of doxorubicin 24 h prior to transfection with the pmeGFP plasmid and either the GFP PO AS Rep oligonucleotide (molar ratio 1/100, grey bars), the GFP dsDNA 700 fragment (molar ratio 1/10, white bars) or with the rAAV-GFPLuc vector (black bars). (B) HEK293T cells were exposed to various doses of phleomycin 24 h prior to transfection with the pmeGFP plasmid and either the GFP PO AS Rep oligonucleotide (molar ratio 1/100, grey bars), the GFP dsDNA 700 fragment (molar ratio 1/10, white bars) or with the rAAV-GFPLuc vector (black bars). (C) Cell cycle distribution of non-treated (left), doxorubicin-treated (middle) and phleomycin-treated cells (right).

Previous reports have shown that DNA damaging agents can increase the efficiency of gene repair [44,45]. In the present study, 3 drugs that activate DNA repair pathways were evaluated for their ability to augment the three different gene repair systems. Initial studies were carried out with the anthracyclin antibiotic doxorubicin (Dox), a commonly used anti-neoplastic agent that has been shown to inhibit the activity of topoisomerase II which results in the generation of single and double-strand DNA breaks. This, in turn, stimulates DNA repair mechanisms. HEK293T cells were preincubated with doxorubicin at concentrations ranging from 0.1 to 300 nM. The cells were transfected with the pmeGFP plasmid and the GFP PO AS Rep oligonucleotide (Table 1). Figure 5A shows that Dox improves the gene correction frequency mediated by ssODN in a dose-dependent manner with an optimum at 30 nM resulting in a 3-fold increase of the proportion of repaired cells. No eGFP positive cells were detected when the non-correcting control oligonucleotide was used (data not shown). Treated and non-treated cells were compared after transfection with the pmeGFP reporter plasmid mixed either with the GFP dsDNA 700 control or correcting fragment, or the rAAV repair vector. No statistically significant increase of the gene correction rate in the presence of Dox was observed with either repair system (Figure 5A).

The other compounds that were tested for their ability to stimulate DNA repair were troxerutin and phleomycin D1. The former compound is a derivative of the natural flavonoid rutin which has been shown to enhance the repair of radiation-induced DNA strand breaks [46], while the latter one is a glycopeptide antibiotic of the bleomycin family that is able to cleave DNA [47]. As with Dox, the cells were incubated with increasing amounts of drug and were then co-transfected with the mutated plasmid and the repair agents. While troxerutin did not improve the repair frequencies (data not shown), phleomycin D1 (PLM) increased the repair efficiency of pmeGFP by about 2 to 3-fold with all three gene repair systems (Figure 5B).

Since the treatment of cells with doxorubicin was reported to alter the cell cycle [48], the cells were assayed for the effect of the experimental conditions on cell cycle. While non-treated cells exhibited a normal cell cycle profile with standard population distributions in the G1, S and G2 phases (Figure 5C), cells treated with 30 nM Dox showed a shift of the population towards the G2 phase (Figure 5C). Cells treated for 24 h with 33 μM of PLM also showed a strong population shift towards the late S/early G2 phase as compared to non-treated cells (Figure 5C).

### Chromosomal gene repair

**Figure 6:**
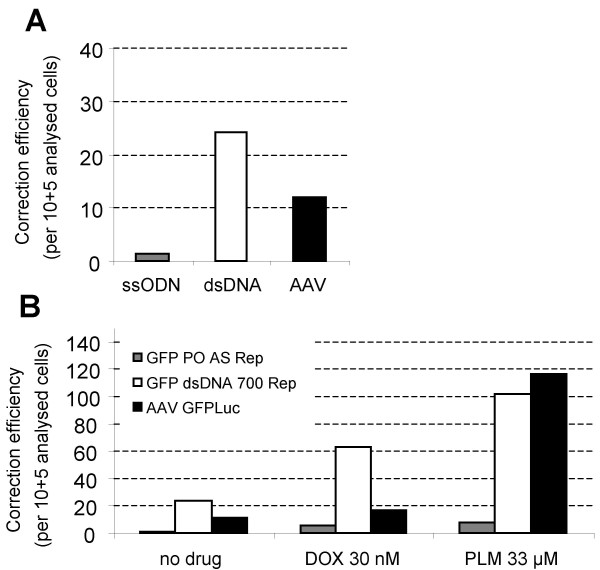
**Chromosomal gene repair assay**. CHO-meGFP-12 cells, pre-incubated (B) or not (A) with doxorubicin (30 nM) or phleomycin (33 μM) were nucleofected with 7.5 μg of GFP PO AS ssODNs or GFP 700 dsDNA fragments or infected with the rAAV-GFPLuc vector (MOI = 300000).

Episomal gene correction assays are useful for evaluating gene targeting systems because they are not time-consuming and they tend to generate higher gene repair frequencies when compared to chromosomal targeting. This is probably due to the high number of targets (plasmids) present during the episomal correction procedure. A better mimic of the therapeutic situation would be to evaluate targeting at the chromosomal level. Therefore, a clone of CHO cells carrying a genomic pmeGFP (CHO-meGFP-12) was generated. The initial repair experiments were conducted with Lipofectamine as the transfection reagent in the episomal assays. However, under these conditions and even with a transfection efficiency ranging between 30 and 40%, no repair events were detected. In contrast, with nucleofection, gene correction frequencies of 2 × 10^-5 ^and 2 × 10^-4 ^were observed with the PO AS Rep oligos and the GFP 700 dsDNA Rep fragments, respectively. No corrected cells were detected when using the control oligonucleotide or the control dsDNA fragment (GFP PO AS Ctr and GFP 700 dsDNA Ctr). Using the rAAV-1-GFPLuc vector, a repair frequency of about 1 × 10^-4 ^was observed (Figure 6A). It should be noted that the efficiency of nucleofection and AAV-mediated transduction of CHO cells are similar (i.e. 7 versus 11% of GFP positive cells), and is the basis for making a comparison between both gene repair systems.

Assessment of correction stability and transmission to daughter cells following exposure to ssODN-, dsDNA- and rAAV was achieved by propagating the cells under selective conditions. Analysis at day 5 and 21 for the proportion of eGFP-positive cells indicated that there is no decline of the proportion of eGFP-positive cells when comparing the proportion at day 5 to day 21 (ssODN: 1.4 × 10^-5 ^and 2 × 10^-5^, dsDNA: 8.1 × 10^-5 ^and 8.4 × 10^-5^, rAAV: 1.2 × 10^-4 ^and 2.19 × 10^-4 ^eGFP-positive cells at day 5 and 21, respectively).

To avoid issues arising from growth and/or survival among the repaired cells clonal expansion was evaluated. Single-cell sorting of the eGFP-positive cells (i.e. repaired cells) was performed at day 5. For each condition, 288 cells were isolated and checked as to their ability to form a colony. About 85% (85%, 83%, 84% and 82% for mock-treated, ssODN-, dsDNA-, and rAAV-treated cells, respectively) of cells not only survived but were able to form colonies. Among the repaired cells and in the presence of neomycin (selection pressure) ~60% displayed GFP fluorescence whereas most of the ~40% non-fluorescent cells became eGFP positive after incubation with 100 nM of trichostatin A (a histone deacetylase inhibitor used to abrogate the silencing of the CMV promoter). Thus, indicating that the apparent loss of expression can be attributed to transgene silencing.

Next, doxorubicin or phleomycin were assessed for their potential to promote gene correction at the chromosomal level. Using the optimized conditions of pre-incubation of the cell cultures with the stimulatory agents previously defined, chromosomal gene correction assays were performed using the CHO-meGFP-12 clone. The results indicate that 30 nM of doxorubicin increases the gene correction frequency by 3-fold both for the oligonucleotides and the DNA fragments, while the AAV-repair was not significantly increased. Phleomycin (33 μM) increased the correction frequency of both, ssODN and dsDNA 700 bp by 5-fold, and the efficiency of rAAV-1 by 10-fold (Figure 6B). Under these conditions, up to 0.1% of repair events occur with the two latter drugs in the overall cell population. It has been previously shown that aminoglycosides can force stop codon readthrough [49]. To exclude the possibility that GFP expression after phleomycin treatment was due to stop codon readthrough, CHO-meGFP-12 cells were nucleofected with a control oligonucleotide in the presence of phleomycin. 5 days after transfection, no GFP expression was detected, indicating that the GFP fluorescence obtained with the repair oligonucleotides was due to gene correction and not to stop codon readthrough.

## Discussion

The aim of the present study was to evaluate the gene editing efficiency of: i- double-stranded DNA fragments of various sizes, ii- different synthetic single-stranded DNA oligonucleotides and iii- a rAAV2/1 vector. To efficiently detect the repair events, two mutated reporter plasmids were generated. The peGFPLucMut expression vector allows for a highly sensitive evaluation of the repair events, while giving the possibility to report the rescued luciferase activity to a percentage of transfected cells. On the other hand, measurement of gene editing at the single cell level, employed assays for targeted modification of a mutated GFP.

The activity of the different repair systems was first evaluated in episomal targets. Experiments where the repair agent was co-transfected with the target gene were useful because the repair frequency was generally 10 to 100-fold higher than gene targeting at the chromosomal level [50]. These assays check whether the oligonucleotides or DNA fragments are able to repair a given mutation and compare repair efficiencies in various cell lines. These experiments also enabled the optimization of transfection conditions as well as provided information about the optimal dose of the repairing DNA. The data in the HEK293T cells was consistent with findings by other groups using other reporter systems, cell lines and/or transfection methods. Among these, we observed that the repair frequency increases with the size of the DNA fragment [7] and that the antisense Luc PS-AS oligonucleotide is more efficient than its sense counterpart in repairing an episomal target [14,50,51]. In addition to testing oligonucleotides with and without 3 phosphorothioate linkages at each end, studies were undertaken to determine whether incorporation of locked nucleic acids (LNAs) into the oligonucleotide sequence can improve the repair frequency. The rationale for including LNA residues was based on their greater affinity to DNA residues than that of DNA itself [42,52]. Thus, it was speculated that LNAs may stabilize the ODN/DNA interactions and result in a more efficient repair. However, none of the three LNA modified ODNs resulted in a higher gene targeting efficiency than the PO or PS oligonucleotide (Figure 2). It is also interesting to note that dsDNA fragments mediated higher repair rates than the oligonucleotides and is in good agreement with results reported by Nickerson and Colledge [50]. Finally, while the rAAV-1 vector gave luciferase values that were comparable to those obtained with dsDNA fragments, the number of repair events in the GFP assay was similar to that observed with oligonucleotides.

Chromosomal repair efficiency was assessed in CHO cells stably expressing the mutated GFP construct. When Lipofectamine was used as the delivery system, no GFP positive cells were detected regardless of the repair system used. Alternatively, nucleofection gave a repair efficiency of up to 0.02% of the overall cell population. Taking into consideration a transfection efficiency of ~10% the actual repair frequency was 0.2% of the transfected cells. This efficiency is comparable to that observed previously by Radecke and co-workers [40] who observed between 0.1 and 0.5% HEK293 cells repaired.

Comparing the different repair systems, roughly the same hierarchy was observed in the episomal and the chromosomal repair experiments. These results are in disagreement with the data published by Nickerson and Colledge [50] who found that dsDNA fragments were more efficient than oligonucleotides in mediating gene repair in the episomal assays while they obtained opposite results in the chromosomal targeting studies. The authors suggest that this difference may be due to the fact that the ODNs have an increased mobility in the cytoplasm due to their small size and thus enter the nucleus more efficiently. While this is likely, it does not account for the episomal targeting where the dsDNA fragments were more efficient. Another possible explanation for the differences observed between the chromosomal targeting in the previous study [50] and the present study is that nucleofection and not lipofection was used as delivery method. Of course, other transfection agents, including cationic polymers may lead to similar results than Nucleofection. Taken together, our results suggest that, when delivered with a comparable efficiency, dsDNA fragments more efficiently repair chromosomal genes than ODNs.

The fact that the AAV-1 vector showed correction frequencies that are lower than those of dsDNA fragments was unexpected given the previously reported chromosomal gene targeting rates for AAV of up to 1% [53,54]. Since AAV-mediated repair frequencies of 0.1% or below have also been reported [55-57] it is possible that there were multiple parameters, such as the target gene, the cell type and the nature of the mutation that influence the efficacy of repair. To the best of our knowledge, previous comparisons between AAV and non-viral repair systems were made indirectly by comparing repair frequencies reported in the literature. Due to the lack of standardized assay conditions, large variations in the rate of gene repair are not surprising and make indirect comparisons somewhat dubious. Furthermore, a recent study showed that homologous recombination is required for AAV-mediated gene targeting [58]. This could explain the similarities in repair efficiency and behaviour observed between AAV and linear DNA fragments in the presence of doxorubicin or phleomycin. However, this does not preclude that there are mechanistic differences between the two repair systems. Moreover, it has been previously shown that linear single- and double-stranded DNA fragments show similar correction efficiencies [5]. This is consistent with episomal repair results using dsDNA and sense and antisense single-stranded DNA fragments of 1000 and 2200 bases generated by asymmetric PCR (data not shown).

In summary, our episomal and chromosomal gene correction studies suggest that, in the absence of drugs such as phleomycin, AAV vectors are less efficient for gene targeting than linear dsDNA fragments. Nevertheless, AAVs remain interesting repair systems due to their ability to transduce a large variety of cells, including primary cells. The efficient nuclear delivery of DNA, even in non-dividing cells, and the recent development of different AAV serotypes that display different patterns of transduction in a diverse array of tissues may be particularly relevant to the use of AAV vectors as gene editing agents. On the other hand, non-viral delivery of dsDNA fragments represents an easier, and potentially safer, way to initiate gene repair than AAV.

Finally, the efficiency of chromosomal gene targeting was significantly increased by treating cells with doxorubicin or phleomycin. It is still unclear whether the enhanced repair was due to the capacity of these drugs to stimulate the DNA repair machinery; however, it is known that dsDNA as well as AAV- and oligonucleotide-mediated repair peaks in the S-phase [59,60]. Since both drugs shift the cell population towards S/G2, the possibility that the increased repair is, at least, in part caused by this population shift in the cell cycle is a probable outcome.

## Conclusion

In conclusion, we would like to emphasize that, despite the apparent low efficiency of targeted gene correction in some cell systems, one can not exclude its use as therapeutic option. In particular when considering *ex vivo *strategies with stem cells [24,61,62]. It has also been shown in mice that a single hematopoietic stem cell is capable of repopulating bone marrow [63]. Therefore, clinical application of gene repair may be feasible when considering treatment for a genetic disease such as X-linked SCID (SCID-X1) where repair of the mutant γc gene in only a small number of cells can be therapeutic [64]. Moreover, by associating targeted gene repair with chromosomal site-specific double strand breaks, that were shown to dramatically increase the correction efficiency of oligonucleotides [65], AAV [57], dsDNA fragments [66,67] and lentiviral vectors [22] gene correction may become a promising therapeutic alternative to conventional gene therapy for the treatment of genetic disorders in the future.

## Methods

### Materials

Dulbecco's modified Eagle's medium (DMEM) with 4.5 g/L glucose and sodium pyruvate, phosphate buffered saline (PBS), Lglutamine, penicillin and streptomycin were purchased from Gibco BRL. Foetal calf serum was purchased from HyClone. peGFPLuc and peGFP-C1 plasmids were obtained from Clontech. Lipofectamine Reagent and Optimem-I medium were purchased from Invitrogen. Doxorubicin chloride (Sigma) and troxerutin (Sigma) dilution stocks were prepared extemporaneously in DMEM and water, respectively. Phleomycin D1 was purchased from Cayla (Invivogen).

### Generation of the mutated reporter constructs

The mutated sequences in peGFPLucMut (6367 bp) and pmeGFP (4731 bp) are shown in Figure 1. The peGFPLucMut plasmid is a derivative of the peGFPLuc plasmid, where one point mutation (T963G, Tyr→*) was introduced into the luciferase portion of the open reading frame (ORF) of the eGFP::Luciferase fusion gene. This mutation generates a premature stop codon upstream of the catalytic site of the luciferase enzyme. The point mutation was introduced using the Quick Change Site-directed Mutagenesis Kit from Stratagene (La Jolla, USA) with the following primers:

5'-TGGCAGAAGCTATGAAACGATAgGGGCTGAATACAAATCACAGAA-3' and 5'-TTCTGTGATTTGTATTCAGCCCcTATCGTTTCATAGCTTCTGCCA-3'. To generate the pmeGFP plasmid, two point mutations (G173A, Trp→* and C177A, Pro→Pro) were introduced, using the same mutagenesis kit as above, into the eGFP-C1 open reading frame of the peGFP-C1 plasmid with the following primers:

5'-CCCGTGCCCTaGCCaACCCTCGTGAC-3' and 5'-GTCACGAGGGTtGGCtAGGGCACGGG-3'.

The first mutation introduces a premature stop codon into the eGFP ORF, whereas the second silent mutation constitutes a tag sequence that distinguishes the corrected (sensitive to MscI restriction endonuclease digestion) from the non-corrected (not digested) plasmids. Plasmids were purified using Macherey-Nagel kits, resuspended in water, aliquoted and stored at -20°C.

### Gene correcting agents

#### Oligodeoxynucleotides

All single-stranded oligodeoxynucleotides were synthesized by Sigma, purified by high-performance liquid chromatography, resuspended in water, aliquoted and stored at -20°C. The sequences of the ssODNs are shown in Table 1. Determination of the melting temperature (Tm) used the following procedure: the LNA-modified and the non-modified oligonucleotides were mixed with a single-stranded complementary DNA fragment (0.05 nmol of each) in a final volume of 25 μl of Absolute Blue Sybr ROX buffer 1× (Thermo Scientific). Samples were first heated to 98°C for 10 min, slowly cooled to 4°C, and then heated from 40 to 95°C at a rate of 0.2°C/30 s. Measurements were taken every 30 s. Assays were performed on a Chromo4 thermal cycler (Biorad) and the Tm was calculated using the accompanying software (ssODN PO: 75.6 ± 0.9°C, PS: 74.9 ± 1.1°C, LNA 1.1 AS: 75.0 ± 1.1 °C, LNA 4.4 AS: 79.7 ± 1.0°C, LNA 4r AS: 78.6 ± 1.0°C).

#### DNA fragments

The dsDNA fragments (195, 494, 980 and 2133 bp) were designed to correct the luciferase mutation and a 732 bp fragment designed to correct the eGFP mutation (see Table 2). The fragments were generated by PCR amplification using DyNAzyme EXT DNA polymerase (New England Biolabs) and primer pairs F200/R200, F500/R500, F1000/R1000, F2200/R2200 and F2200/R700, respectively (see legend of Figure 1). After a first round of PCR amplification (20 cycles), the fragment of interest was gel-purified to eliminate the plasmids used as templates. A second round of PCR amplification was then performed (25 cycles), and the PCR products were subsequently purified, resuspended in water, aliquoted and stored at -20°C. The correcting fragments (dsDNA Rep) were generated using the peGFPLuc and the peGFP-C1 plasmids as templates whereas the peGFPLucMut and pmeGFP plasmids were used as templates to generate the non-correcting control fragments (dsDNA Ctr).

#### Production, purification and titration of the rAAV vectors

**Figure 7:**
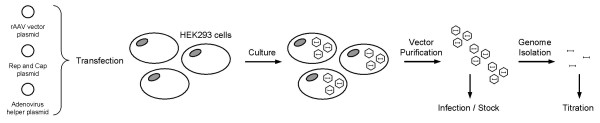
**Representative scheme of the rAAV production procedure**. Recombinant AAV vectors were generated using a three-plasmid transfection protocol. Briefly, HEK293 cells were tri-transfected using polyethylenimine (PEI 25 kDa; Aldrich) with the helper plasmid carrying adenovirus helper functions, the packaging plasmid expressing the *rep *and *cap *genes and the rAAV vector plasmid containing the vector genome sequence. Three days after, cells were harvested and lysed to release the recombinant vectors from the producer cells. Samples were centrifuged at 1500 g for 15 minutes at 4°C and supernatants were treated with 25 U/ml benzonase for 30 minutes at 37°C, centrifuged at 10000 g for 20 minutes at 4°C. Then, one volume of cold saturated ammonium sulfate was added to supernatants and samples were incubated 1 h on ice and centrifuged at 12 000 g for 30 minutes at 4°C to precipitate the recombinant viral particles. rAAVs, resuspended in PBS with Ca^2+ ^and Mg^2+ ^were then purified by 2 rounds of ultracentrifugation on isopycnic CsCl_2 _gradients. The vector-containing fractions were then pooled and desalted by dialysis against sterile PBS supplemented with Ca^2+ ^and Mg^2+^. Purified recombinant vectors were aliquoted and stored at -80°C. For the titration of the physical particles, an aliquot was treated with DNAse I for 10 minutes at 37°C to eliminate residual DNA. Then, the sample was treated with proteinase K to degrade the capsids and to release the viral DNA. Viral genome titer of the sample was then determined by real-time qPCR.

Pseudotyped AAV-2/1 vectors were generated using the three-plasmid transfection protocol described by Rivière *et al. *[68] with minor modifications. Representative scheme and details about the production, the purification and the titration processes of the rAAV vectors are given in the legend of Figure 7. For our purpose, the productions were realized with the pXX6 adenovirus helper plasmid, the pLT-RC02 packaging plasmid expressing the *rep *and *cap *genes and the pSMD2-eGFPLuc or the pSMD2-eGFP plasmid. The latter drive the synthesis of the viral genome composed of the homologous sequences used to target the mutated genes flanked by the AAV-2 inverted terminal repeats (ITR). The pSMD2-eGFPLuc plasmid was generated from the pSMD2-Luc plasmid [69] into which the NheI-XbaI 2.4 kb fragment of peGFPLuc (which contains the eGFPLuc ORF) was subcloned between the two inverted terminal repeats (ITRs) of the XbaI-XbaI 4 kb fragment-deleted pSMD2-Luc plasmid (total viral genome size: 2.8 kb). The pSMD2-eGFP plasmid was generated from pSMD2-eGFPLuc by excising the BglII-XbaI 1.6 kb fragment containing the Luc ORF. Note that Hirata and co-workers [20] have previously shown that with a size of 2845 bases, viral genomes are only present as monomers in rAAV. Since the viral genome containing the 2.4 kb peGFPLuc fragment has a size of 2.8 kb, we can assume that our AAV vectors are single stranded.

### Cell culture

All cell lines were cultured at 37°C, 5% CO_2_, and 100% humidity in DMEM supplemented with 10% heat-inactivated foetal calf serum, 2 mM L-glutamine, 100 U/ml penicillin and 100 ug/ml streptomycin (standard medium). Only cells that were mycoplasma free and less than 15 passages were used.

### Generation and characterization of the CHO-meGFP-12 cell clone

CHO-K1 cells were transfected using Lipofectamine reagent mixed with StuI linearized pmeGFP plasmids. The cells were cultivated for 3 days in standard medium before the addition of geneticin sulfate (600 μg/ml) to select the stably transfected cells. Two weeks after transfection, geneticin-resistant colonies were harvested and cloned with a cell sorter. Clone CHO-meGFP-12 was selected for the repair experiments. The copy number of the integrated eGFP mutated expression cassette has been determined by qPCR using Taqman probes directed against the CMV promoter and the 3' region of the eGFP gene. The clone CHO-meGFP-12 contains 20 non-concatemerized copies of the eGFP mutated expression cassette. The absence of concatemers was demonstrated by nested PCR amplifying all the possible forms of concatenation.

### Gene correction assays

Episomal gene correction assays were performed as follows: plasmid DNA- and the gene repair agent (ssODN and dsDNA)- complexes were generated separately in Optimem-I medium with Lipofectamine Reagent using a cationic lipid/DNA (w/w) ratio of 4:1. Exponentially growing HEK293T cells were harvested and 10^5 ^cells were seeded into a 24-well plate containing 1 ml of standard medium supplemented or unsupplemented with DNA repair stimulating drugs (e.g. doxorubicin, troxerutin or phleomycin). The next day, culture medium was discarded, cells pre-incubated with the repair activating drugs, were washed once with standard medium. The transfection mixture was then added in a final volume of 0.5 ml/well. After a 3 h incubation, the transfection medium was removed and fresh standard medium was added until analysis. When rAAV vectors were used as repair agent, the culture medium was removed one hour after the transfection step (which allowed for the delivery of the mutated reporter construct) and replaced by the infection mixture consisting of diluted rAAV-2/1 vectors (0.25 ml medium/well). The infection mixture was removed after 12 h and replaced with fresh medium. Analyses of the episomal repair frequencies were performed 48 hours after the transfection/infection.

Chromosomal gene correction experiments with ssODN and DNA fragments involved seeding 2 × 10^6 ^cells into a culture flask in medium that was either supplemented with drugs or unsupplemented. After an overnight incubation, the cells were washed twice with PBS, detached with trypsin-EDTA 0.05% and then resuspended in standard medium before centrifugation at 200 × g for 10 minutes. The cell pellet was resuspended in Solution T (from Amaxa Biosystem) at a concentration of 1.2 × 10^7 ^cells per ml. A 100 μl aliquot of the cell suspension was slowly mixed with 7.5 μg of the gene correction agent before nucleofection with program U-23 of the Nucleofector II device (Amaxa Biosystem). After nucleofection cells were immediately seeded into 10 cm dishes containing pre-warmed standard medium and were grown under selection (600 μg/ml Geneticin) for five days before analysis. When using rAAV vectors for chromosomal gene correction, exponentially growing cells were harvested and seeded into a 24-well plate at a density of 3 × 10^4^/well without or with drugs. The following day, the cells were preincubated with DNA repair activating drugs, washed once with standard medium and then grown in infection mixture (0.25 ml/well final volume). After a 12 h incubation, the infection medium was removed, and replaced with fresh medium and cells were grown in the presence of 600 μg/ml Geneticin for four days before analysis.

### Flow cytometry analysis

Cells were analysed by flow cytometry after they were detached with trypsin-EDTA 0.05% and diluted in 20 volumes of standard medium. The analysis was carried out on a Facscalibur flow cytometer (BD; Becton, Dickinson and Company) using the software CELLQuest, version 3.1f (BD). Quantification of gene repair rates was achieved using 50 000 morphologically intact cells to allow detection of eGFP positive cells. The respective repair rate was calculated as the ratio of morphologically intact eGFP-positive cells to all morphologically intact cells. The proportion of eGFP-positive cells is expressed as a percentage and the values are the means of duplicates.

### Cell cycle analysis

Cells were harvested with trypsin-EDTA (0.05%), washed twice with PBS and fixed in 70% cold ethanol. The fixed cells were then stored for 24 h at 4°C before PI staining (propidium iodide at 40 μg/ml, DNAse-free RNAseI at 20 μg/ml and 0.1% sodium dodecyl sulfate in PBS) at 37°C for 3 h. The proportion of cells in each phase of the cell cycle is expressed as percentage and all values are the means of duplicates.

### Luciferase assay

The luciferase assay was performed as previously described [70]. Background luciferase activity was subtracted from each value. The protein content of transfected cells was measured by Bradford dye-binding using the Biorad protein assay. Luciferase activity levels are expressed as light units per 10 second per milligram of protein and the values are the means of duplicates.

## Authors' contributions

XL designed and carried out the experiments, interpreted the data and co-wrote the manuscript. DS provided intellectual and financial support and helped to draft the manuscript. OD participated in the conception of the study and helped to draft the manuscript. AK conceived the study and participated in its design and coordination, and co-wrote the manuscript. All the authors have read and approved the final manuscript.
